# Synthetic and systems biology for microbial production of commodity chemicals

**DOI:** 10.1038/npjsba.2016.9

**Published:** 2016-04-07

**Authors:** Victor Chubukov, Aindrila Mukhopadhyay, Christopher J Petzold, Jay D Keasling, Héctor García Martín

**Affiliations:** 1 Joint BioEnergy Institute, Emeryville, CA, USA; 2 Biological Systems and Engineering Division, Lawrence Berkeley National Laboratory, Berkeley, CA, USA; 3 Department of Chemical & Biomolecular Engineering, University of California, Berkeley, CA, USA; 4 Department of Bioengineering, University of California, Berkeley, CA, USA

## Abstract

The combination of synthetic and systems biology is a powerful framework to study fundamental questions in biology and produce chemicals of immediate practical application such as biofuels, polymers, or therapeutics. However, we cannot yet engineer biological systems as easily and precisely as we engineer physical systems. In this review, we describe the path from the choice of target molecule to scaling production up to commercial volumes. We present and explain some of the current challenges and gaps in our knowledge that must be overcome in order to bring our bioengineering capabilities to the level of other engineering disciplines. Challenges start at molecule selection, where a difficult balance between economic potential and biological feasibility must be struck. Pathway design and construction have recently been revolutionized by next-generation sequencing and exponentially improving DNA synthesis capabilities. Although pathway optimization can be significantly aided by enzyme expression characterization through proteomics, choosing optimal relative protein expression levels for maximum production is still the subject of heuristic, non-systematic approaches. Toxic metabolic intermediates and proteins can significantly affect production, and dynamic pathway regulation emerges as a powerful but yet immature tool to prevent it. Host engineering arises as a much needed complement to pathway engineering for high bioproduct yields; and systems biology approaches such as stoichiometric modeling or growth coupling strategies are required. A final, and often underestimated, challenge is the successful scale up of processes to commercial volumes. Sustained efforts in improving reproducibility and predictability are needed for further development of bioengineering.

## Introduction

Although the synthesis of urea by Wohler in 1828^1^ established that biological entities are not radically distinct from purely physical ones, we are still unable to design and engineer biological systems with the same ease and precision with which we design physical ones (e.g., cell phones, automobiles or jet planes). Whether biological systems can be understood as a mechanistic composition of physical parts is a fundamental philosophical and scientific problem, epitomized by the understanding of the brain and the emergence of consciousness.^2^ Furthermore, engineering of biological systems has emerged as one of the most exciting recent technologies. Applications for human health include tumor-killing bacteria, *in vivo* diagnostics, engineered viruses and immune cells that target specific disease cells, synthetic drug delivery vectors, and even completely synthetic tissues.^3–5^ Environmental applications include microbes that sense, report, and degrade toxic chemicals,^6,7^ while synthetic biology has the capability to produce a variety of chemical products ranging from therapeutics to plastics and biofuels.^8–10^

This last application of microbial bioengineering to produce biofuels and other commodity products has attracted significant attention due to its potential to mitigate climate change, reduce society’s reliance on fossil fuels, and improve energy security. Renewable chemical production is critical to achieving these goals, particularly given the needs of the growing populations of emerging economies.^11^ In 2004, a US government report energized the metabolic engineering community by providing a list of targets that would have a transformative impact on biomanufacturing,^12^ if they could be made using microbial platforms from sustainable carbon sources. Efforts in the last decade, aided by systems and synthetic biology, have succeeded in producing a variety of these and other products, typically at titers of μg/l to mg/l. However, production of only a few compounds, such as 1,3-propanediol,^13^ 1–4-butanediol,^14^ and artemisinin^15^ has reached commercial scale, attesting to the difficulty of this process.^16^ Improving the yield, titer, and productivity of microbial processes to enable commercialization requires detailed manipulation of microbial physiology, stress response, and metabolism, with particular emphasis on carbon and energy balance. In the case of bulk commodities, where commercial viability requires capturing every available carbon atom and eliminating every unneeded ATP sink, metabolic engineering requires a systems biology^17^ approach where the interaction of the exogenous pathway with host metabolism can be explicitly considered and understood.

**Figure 1:**
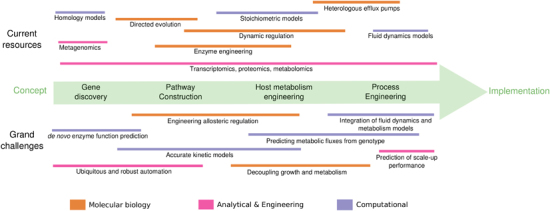
The process of bioengineering strains for commodity chemicals from initial concept (target molecule selection) to scale up (process engineering and implementation), along with a selection of tools applicable to each step and the grand challenges that need to be met. The lines, colored according to the type of tool/challenge, indicate which parts of the process the tool or challenge applies to (e.g., dynamic regulation can be used for pathway construction but also for control of toxic intermediates that affect host metabolism). Two of these tools and challenges are highlighted in the following figures (Figures 2 and 3). In spite of the linear diagram chosen to represent them, it must be understood that the process is rarely sequential: e.g., very often problems in engineering the process for scale up force researchers to go back to pathway construction and make significant changes.

In this review, we present the difficulties of taking a microbial production process from conception to commercialization along with the tools that can be used to address some of the challenges and gaps in our knowledge and engineering capabilities (Figure 1). At a time when a significant acceleration of biological engineering is possible,^18^ but requires an influx of talent from a variety of engineering and physical sciences,^19^ this review presents the challenges that we think future metabolic engineers and synthetic biologists may want to address for fastest development of the field.

## Pathway design

### Target molecule selection

The power of using microbial processes for chemical production is twofold: first, renewable carbon sources can serve as substrates, and second, the range and specificity of molecules that can be made biologically surpasses that of synthetic chemistry. The impressive selectivity of biological systems allows precise control over the chemical features of the final product, such as chirality and positioning of functional groups. This large range of molecules means that, while current efforts focus largely on well-characterized molecules, the most exciting applications will come when new molecules with unique chemical properties give rise to new materials, polymers, and fuels. Given the challenge in predicting chemical and material properties *de novo*, selecting promising future targets for biological production will require extensive collaboration between chemists, engineers and biologists, and the development of tools able to predict the bulk properties of materials composed of novel molecules.

Because of the difficulties of entering new chemical markets, most biological production has focused on molecules with large existing markets. Initial efforts focused on high-price, low-volume markets such as pharmaceuticals, where biological production could make an immediate and obvious impact.^20^ As interest has developed in high-volume, low-cost markets such as biofuels, economic considerations have become paramount, and development of a new project must begin with an analysis of the potential of process commercialization. This multi-disciplinary effort unites very disparate sets of knowledge. On one hand, the commercial potential of the molecule must be assessed. This potential depends not only on chemical properties and known performance but also on highly volatile market data: from the feedstock price (e.g., corn, sugar cane, or lignocellulose) to the current molecule price, both of which are tightly coupled to other market and political processes such as fluctuating oil prices, economic cycles, and uncertainties in government regulatory policies. On the other hand, the feasibility of producing the desired molecule biologically must be assessed, something which is still extremely difficult in the current state of bioengineering. Adding to these difficulties is the complexity of predicting the extra economic costs derived from scaling up production (described below) and downstream processing (e.g., molecule extraction and purification). All these factors make target molecule selection the least systematic part of the metabolic engineering process.

Due to the difficulty of assessing economic viability, metabolic engineers typically resort to a variety of available reports indicating high-value targets or intermediates as indicated by experts in the field,^12,21–24^ and rely on their own personal expertize to judge which of them can be biologically tractable. Currently, this approach works satisfactorily because troubleshooting the bioengineering effort takes significantly more time than choosing the target. However, as it becomes possible to biologically produce new types of molecules more efficiently,^18^ it would be desirable to create tools that combine technoeconomic analysis with market needs based on chemical properties, as well as systematic biological feasibility estimates and scale-up considerations.

Even in the cases where biomanufacturing has not achieved economic cost-competitiveness, it can help improve the sustainability of energy sources and other chemical commodity products. Despite the recent drop in oil and gas prices, 195 nations committed to reducing greenhouse gas emissions at the 2015 negotiations in Paris,^25^ and biomanufacturing provides a path toward a decarbonized, more sustainable economy. As countries across the world work to integrate the full social and environmental cost of fossil-derived fuels and products, bio-derived products will become increasingly attractive. The biomass-derived biofuel farnesene,^26^ e.g., is used to power part of the bus fleet in Sao Paulo, one of Brazil’s largest cities, even though the prize is higher than standard diesel: the city has pledged to reduce fossil diesel fuel use by 10% every year and are willing to subsidize higher prices to achieve this goal. Similarly, in the United States the establishment by congress of a renewable fuel standard^27^ that requires the use of 36 billion gallons of renewable fuel by 2022, provides the necessary incentives to pay for the offset in greenhouse gas emissions that renewable fuels provide as their main competitive advantage.

### Gene discovery and pathway construction

Once a target is selected, the best production pathway needs to be identified. Historical methods for pathway discovery were limited to insights from experts in the field and manual gene selection that was informed by little functional data. More recently, computational methods based on biochemical reaction databases and genome mining have automated the process and implemented scoring methods to prioritize pathway selection. Developments in next-generation sequencing technology along with bioinformatic analyses have revolutionized gene discovery methods for metabolic engineering by providing a vast resource of genomic data to query for target genes. Many tools are available to use this data to identify biosynthetic gene clusters^28,29^ or select pathways based on homology-predicted enzyme function retro-synthesis of products.^30–33^ Yet, identification of gene function through homology comparison is quite poor for genes with unique functions, and functional screens are needed to identify specific steps in a desired pathway. Furthermore, experimental validation of predictions from these bioinformatic tools remains limited, so the success rate is unknown. Even when there is high confidence in the gene function, most retro-synthesis tools do not have the data needed to identify the organism containing the gene version that would yield optimal protein production and activity, so multiple gene variants must be tested to find the optimal enzyme for a given reaction step.

Pathway construction methods have been revolutionized by the advent of next-generation sequencing and affordable *de novo* DNA synthesis coupled with standardized expression vectors and genomic integration methods. Although traditional pathway construction is iterative and specific to the desired construct, these methods open the door to combinatorial pathway construction of vast libraries consisting of host genetic contexts, open reading frames, and/or protein expression variants. By producing a deep library of different pathway constructs a researcher can select a subset of strains either from pre-determined parameters or from design-of-experiment methods for subsequent testing and analysis. For systems with a well-defined high-throughput screen,^34^ selection, or biosensor system, large (10^7^–10^10^) combinatorial libraries can be constructed, analyzed, and the identity of the best-performing strains can be determined by sequencing. This data driven workflow for strain construction and selection redistributes resources toward experimental design and data analysis efforts to reach production goals. Although these capabilities are ready to be deployed with traditional model systems such as *Escherichia coli*, they can also be used for many other microbes where synthetic biology and genetics are rapidly maturing, such as pseudomonads,^35^ autotrophs such as cyanobacteria,^36^ and *Ralstonia eutropha.* ^37^ The challenges and opportunities to improve pathway construction, especially the automation of DNA design and construction, are described in several recent reviews^38–40^ and research efforts,^41,42^ and are not reviewed here.

## Pathway optimization

### Characterizing enzyme expression by proteomics

Merely introducing the genes into a host provides no guarantee that the synthetic pathway will function effectively. Often, the first troubleshooting steps involve ensuring expression of the introduced genes. Although reverse transcription–PCR or RNA sequencing can verify transcription, defects in translation or protein stability are more common, necessitating protein-level analysis. Consequently, development of rapid and accurate high-throughput assays to monitor protein expression can dramatically accelerate engineering efforts. Protein analysis is frequently achieved via immunoblot assays because they are selective and easily analyzed in parallel. However, assaying many different pathway proteins in the same strain can be challenging and quantification is often inaccurate and difficult to reproduce. Mass spectrometry-based proteomic methods have risen in popularity, as they can identify and quantify thousands of proteins. Relative quantification of proteins between different engineered strains is typically based on targeted proteomic methods via selected-reaction monitoring mass spectrometry, and takes advantage of isotopically labeled substrates or chemical tags (e.g., iTRAQ^43^ or TMT^44^) for accurate protein quantification.

The first reported targeted proteomics study of engineered *E. coli* identified protein-associated bottlenecks in the mevalonate pathway used to produce the sesquiterpene amorpha-4,11-diene.^45^ Here, levels of two proteins, mevalonate kinase (MK) and phosphomevalonate kinase (PMK), were particularly low. To overcome these bottlenecks the *MK* and *PMK* genes were codon-optimized for translation in *E. coli* and also expressed from a stronger promoter. These changes led to significant improvements in the protein levels and amorpha-4,11-diene production.

Aside from identifying pathway bottlenecks, comparative proteomics is also commonly used to quantify native host proteins, which can identify cellular stresses and perturbations to host metabolism. These may be based on targeted methods as above, or untargeted shotgun proteomic analyses via many “label-free” techniques.^46^ A number of recent studies have characterized cellular responses to potential biofuel products^47–51^ and identified metabolic sinks that impact carbon utilization.^52^ Such analysis will become even more critical as heterologous pathways increase in efficiency, demanding more cellular resources and imposing stresses and constraints on host metabolism. As current state-of-the-art methods still require relatively long chromatographic separation times (tens of minutes to hours) and are performed by using nano-flow chromatography, they are susceptible to variations in sample preparation and chromatography. As a result, these methods are not immediately suitable to high-throughput analysis and method development will need to address both throughput and data quality.

### Optimizing expression levels

Product yield optimization can be achieved, in part, through fine-tuning of exogenous pathways in order to maximize the flux through the introduced pathway. Nonetheless, it is not straightforward to know in advance which pathway designs will produce the highest production. Ideally, the optimal expression level for each enzyme would be guided by accurate kinetic models of the pathway.^53–57^ However, this approach is hampered by a variety of challenges, such as lack of reliable data for enzyme activity and substrate affinity parameters, *in vivo* protein quantification, and the effects of activators and inhibitors. In spite of these hurdles, approaches that parameterize the kinetic model based on a subset of data via ensemble modeling^58,59^ have been successfully implemented, for instance to improve neurosporene productivity. In other studies, kinetic models have been used to pinpoint rate-limiting steps for hydrogen production.^60^ In the absence of accurate kinetic models, heuristic statistical approaches have been successfully used to guide product yield increases. Typically, predictions are extrapolated from data obtained from a relatively small (compared with the full combinatorial space) set of pathway designs. In this vein, Ajikumar *et al.* ^61^ improved taxadiene production by dividing the pathway into different modules and performing a multivariate search, an approach that was expanded by the use of a linear regression model^62^ for amorphodiene production. A linear regression model was also used in order to relate protein expression levels to the production titers of the four primary products in the violacein pathway.^63^ Alonso-Gutierrez *et al.* ^64^ used quantitative proteomics data and principal component analysis to increase limonene and bisabolene production, while George *et al.* ^65^ used correlations between proteomic and metabolomic data to derive a qualitative model of the mevalonate pathway for isopentenol production. However, it must be recognized that for any of these methods, even if the desired expression level for maximum production is known, it is a non-trivial task to design the right pathway to obtain it.

In addition, optimizing expression levels is crucial if overexpression of key proteins imposes a large cellular burden due to toxicity. Although most observations of toxicity caused by protein expression relate to protein function, certain categories of proteins are more directly toxic to the cell. Examples include transport proteins that are important both for the import of carbon sources or nutrients, as well as exporters that are required to secrete final products or exclude harmful by-products left over from substrate processing.^66,67^ Overexpression of membrane proteins commonly leads to growth inhibition, likely due to altered membrane physical properties and limited expression and translocation of key proteins such as electron transport chain components, which has significant downstream impact on central carbon metabolism.^68^ These tradeoffs must be considered when expressing multiple membrane proteins during strain engineering.^69^ Protein expression burden can be addressed using directed evolution to obtain variants that perform efficiently within the range of expression that the strain accommodates^70^ or by controlling protein expression more dynamically in response to conditions under which the tolerance phenotype is required.^71,72^

### Toxicity of pathway intermediates and the role of dynamic regulation

Natural metabolic pathways have evolved intricate mechanisms to avoid the formation of potentially toxic intermediates.^73^ As external pathways and enzymatic reactions are introduced into cells, it is not surprising that a number of the pathway intermediates with undesirable negative effects on cell growth and target production may accumulate. Even when the pathway is entirely native, metabolic engineering involves increasing flux by several orders of magnitude, meaning that imbalances in enzyme activity levels may lead to larger fluctuations in metabolite concentrations than would be found naturally. This has been observed in several pathways prominently used in metabolic engineering in the last decade. For example, the mevalonate-based isoprenoid pathway derived from *Saccharomyces cerevisiae* has been used for the production of anti-malarial drug artemisinin, as well as a number of biofuel molecules. Two intermediates in this pathway are known to cause cellular growth inhibition. One is 3-hydroxy-3-methylglutaryl-coenzyme A (HMG-CoA), which leads to a feedback regulation in the fatty acid biosynthesis pathway due to its similarity to malonyl-CoA.^74^ The second is downstream of the main monomer generated from this pathway, isopentenyl pyrophosphate. Isopentenyl pyrophosphate itself is toxic to the cell, but the subsequent prenyl diphosphates geranyl pyrophosphate (GPP) and farnesyl pyrophosphate (FPP), are progressively more toxic. The toxicity of these compounds was evaluated using strains that accumulate isopentenyl pyrophosphate, GPP, and FPP, and the accumulation of FPP was found to be highly growth inhibitory in *E. coli.* ^75^ Accumulation of prenyl pyrophosphates is also inhibitory in other metabolic engineering strain platforms,^76–78^ and has required optimization.

Alleviation of intermediate toxicity can be achieved by rebalancing the expression of the pathway enzymes, in particular by increasing the expression of enzymes downstream of the intermediate.^78^ However, careful static manipulation of the expression of every pathway gene is difficult, and a more systems biology approach is to mimic the circuits built by nature to address this challenge. Circuits in which a metabolite regulates its own production or consumption can afford very tight control over the intracellular concentration. Such an approach was taken in Dahl *et. al.,* ^79^ where a native *E. coli* promoter that was negatively regulated in response to FPP stress was used to regulate the upstream *atoB* gene, while a positively regulated promoter was used to drive the *ads* gene downstream.

The potential applications of dynamic regulation go beyond the relief of intermediate toxicity. Ideally, the activity of every enzyme would be regulated by the balance of its substrates and products, and overall pathway activity would be regulated by signals corresponding to sufficient carbon and energy availability, ensuring that production does not interfere with cell viability. The potential of this approach for improving production was demonstrated more than a decade ago^80^ by using promoters regulated by the cellular metabolic state to control the rate-limiting steps of the pathway. An analogous approach was also used to optimize fatty acid ethyl ester production in *E. coli*. Here the FabR transcriptional factor that interacts with a modified promoter only in its apo form was used to regulate expression of the pathway genes, resulting in optimal gene expression only in the presence of free fatty acids, again resulting in greater and more stable production levels.^81^ Since then a variety of regulatory motifs have been developed^82^ with one of the most advanced being a tiered control that could integrate five separate cues into one final output.^83^ Such regulatory motifs will be required as we develop strains that can withstand a range of perturbations and dynamically optimize production in the growth modes needed for industrial production.

**Figure 2:**
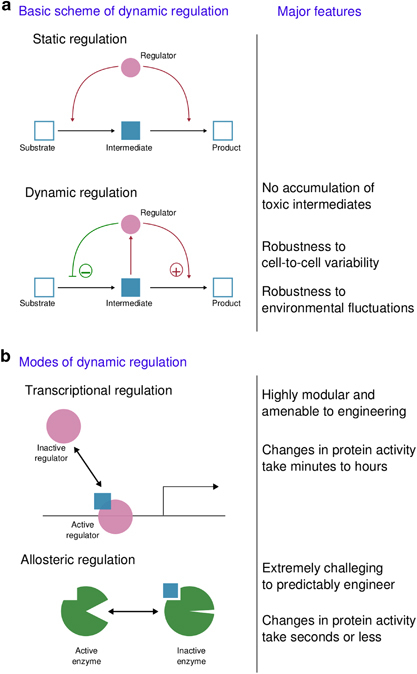
Dynamic regulation for metabolic pathways displays significant advantages with respect to static regulation. The largest challenge in this area involves using allosteric mechanisms for this regulation. We envision future cells that can be programmed to respond quickly to internal and external signals, and demonstrate phenotypes that are as robust as the metabolism of naturally evolved organisms. (**a**) Shows the basic scheme of dynamic regulation as opposed to static regulation. (**b**) Explains two possible modes of dynamic regulation.

Almost all instances of dynamic regulation developed to date rely on transcriptional regulatory circuits, since transcriptional control is highly modular and well characterized. However, transcriptional control circuits have serious limitations, mostly due to two closely related factors: first, metabolic fluctuations occur on time scales much faster than the transcriptional response, and second, transcriptional activation is largely irreversible, as accumulated protein must either slowly be diluted over a long generation time, or be degraded and resynthesized at a great metabolic cost. A significant challenge will be to engineer circuits based on allosteric regulation (Figure 2), or other fast and reversible protein–metabolite interactions. These circuits would mimic natural control and allow enhanced regulation of metabolic processes. We envision future cells that can be programmed to respond quickly to internal and external signals, and demonstrate phenotypes that are as robust as the metabolism of naturally evolved organisms.

## Engineering of host metabolism

### Finding optimal flux distributions

In parallel to pathway optimization, manipulating host metabolism to direct as much flux as possible into the desired pathway is a critical aspect of metabolic engineering. An engineered pathway must operate in the context of the rest of cellular metabolism, which acts as both a blessing and a curse. On one hand, metabolic engineers can rely on innate cellular pathways for sugar catabolism and generation of not only the building blocks that form the starting point for the desired pathway, but also energy and redox cofactors such as ATP, NADH, or NADPH. Cells have machinery to sense an increased demand for these cofactors and adjust their metabolism accordingly,^84^ but this metabolic control is optimized for growth and survival, making host engineering necessary to optimize the cell for production. On the other hand, an overwhelming demand for carbon and energy from the engineered pathway may impose an insurmountable burden for the cell, which has to carry out a variety of other processes,^85^ necessitating significant systems-level approaches in host engineering that rely on large-scale models of cell metabolism.

Stoichiometric genome-scale metabolic models are now frequently used for considering the entire metabolic network and understanding how alterations in central pathways propagate to the rest of cellular metabolism.^86^ The first and simplest use of stoichiometric models (genome-scale or not) is finding the maximal theoretical yield of product and the distribution of fluxes in central metabolism that leads to the optimal yield.^87^ Algorithms such as OptStrain^88^ can search reaction databases and find additional reactions that could be added to the network to improve theoretical yield. Second, stoichiometric models can be used to predict the effects of gene deletions on the flux distribution of the considered strain. These algorithms rely on some heuristic assumptions, typically that the distribution will be as close as possible to the wild-type (e.g., MOMA,^89^ ROOM,^90^ RELATCH^91^) or that some aspect of the wild-type distribution, such as a specific flux ratio, will be conserved (PFF^92^). Despite the necessary heuristic assumptions, these methods have been quite successful and have found applications in metabolic engineering, such as for dihydroartemisinic acid production.^93^ When product yield optimization is the main goal, a third use involves identifying the fluxes that are required to change in order to achieve the desired product yield increase (e.g., OptForce^94–96^ and FSEOF^97^). The corresponding genes are attractive targets for overexpression or knockdown, even though there is no guarantee that manipulating their expression will lead to a corresponding change in flux. For the last two use cases, a critical input is the reference (wild-type) flux distribution, which is often obtained *in silico* by assuming maximal biomass yield (Flux Balance Analysis). However, since results may significantly depend on this reference flux profile and it is not clear that the growth maximization principle is universally applicable (particularly for bioengineered reference strains), it is desirable to obtain this reference flux distribution more accurately through ^13^C tracing experiments.^98–100^ The combination of these labeling experiments with genome-scale models^101,102^ provides a seamless route to use this information to improve predictions. A final use for stoichiometric models is designing “growth-coupling” strategies wherein maximization of biomass yield actually forces flux through the desired production pathway,^103–105^ as explained in detail below.

Next-generation genome-scale models expand current metabolic models by taking into account the metabolic investment in expressing proteins,^106^ or by coupling simulations for all the constituent cellular processes.^107^ It remains to be seen whether, for metabolic engineering purposes, the extra predictive power compensates for the added complexity in these modeling frameworks.

### Balancing of required cofactors

Highly reduced products such as biofuels often rely on reductive anabolic pathways, which typically require NADPH as an electron donor. As most typical microbes generate NADH during glycolysis, the stoichiometric analysis described above often identifies the imbalance between these cofactors as a major problem. Alternative catabolic pathways may be available—a popular method includes deletion of the phosphoglucoisomerase (*pgi*) gene, forcing glucose catabolism through the NADPH-generating pentose phosphate pathway,^108^ while genome-scale stoichiometric models can identify other less intuitive genetic changes likely to increase NADPH production.^109,110^ A recent successful example focused on overexpression of Entner-Douderoff pathway enzymes from *Zymomonas mobilis* in *E. coli.* ^111^

An alternative approach is to modify the specificity of an enzyme to accept the alternative redox cofactor, which can often be done by a rational, structure-guided approach. Javidpour *et al.* ^112^ modified one step of the fatty acid biosynthesis pathway to accept NADH instead of NADPH, and found a concomitant increase in the production of either free fatty acids or long chain methyl ketones. Cofactor specificity could potentially also be modified in central metabolism, to produce NADPH instead of NADH, however, successful examples of this are relatively sparse. One key consideration is the thermodynamic constraints: since most organisms keep NADPH/NADP+ ratios significantly higher than NADH/NAD+ ratios, more energy is required to regenerate NADPH than NADH. Thus, algorithms based on stoichiometry to find optimal points in metabolism for altering cofactor specificity^113^ need to consider whether sufficient thermodynamic driving force can be maintained.^114^

### Growth coupling

Although identifying the optimal flux distribution may guide some genetic changes that bring the metabolic network closer to the optimal state, there is generally no guarantee. Host cells are adept at finding new ways to reroute metabolism and avoid the fitness cost that underlies shifting resources away from growth to production of the targeted molecule. A powerful approach, which can also be guided by stoichiometric analysis, is to engineer a cell in which production is growth coupled, i.e., cellular growth by necessity produces the desired compound as a by-product. This is the situation in anaerobic fermentation, where excess reducing equivalents from sugar catabolism are forced to be secreted as products like ethanol and butanol. The requirement is that the pathway between sugar and product be redox balanced and produce other by-products necessary for cell growth, typically ATP from substrate-level phosphorylation.

This basic strategy was implemented in a number of studies. The first study in *E. coli* eliminated ethanol and lactate secretion pathways and imposed anaerobic growth conditions, leaving succinate production as the main electron sink.^115^ After several generations of evolution, the authors were able to obtain higher succinate titers than any previously reported. In a study by Shen *et al.,* ^116^ the succinate sink was also eliminated and a heterologous pathway for n-butanol production was inserted as the sole electron sink, and a similar strategy was employed for n-hexanol^117^ and lactate production.^118^ When the pathway is not perfectly redox balanced, a more oxidized substrate can be used, such as gluconate instead of glucose.^119^ The net ATP gain is critical in these schemes, making the recent development of fatty acid biosynthesis by reverse β-oxidation^120^ very exciting. Fatty acid biosynthesis combined with chain termination enzymes can be used to produce a large variety of long-chain molecules with great potential for use as fuels and chemicals,^121^ but requires all of the ATP produced from glycolysis for each extension. However, in reverse β-oxidation, no ATP is required after the first extension step, making growth coupling possible. A careful investigation of the potential of this method is provided by Cintolesi *et al.* ^122^

Many algorithms to identify other potential growth coupling schemes based on genome-scale stoichiometric models of metabolism have been developed,^103,105,123,124^ but success stories of growth coupling that are not redox based are sparse. Many examples exist of genetic screens for enzymes that complement auxotrophy and these have been used to select for functional enzymes in amino acid biosynthesis^125^ or novel pathways to isoprenoids.^126^ However, in these cases the selection pressure disappears after a minimum production level, and a stronger effect can be obtained by screening for resistance to an inhibitor.^127^ Although these assays allow for growth-based screening or short-term evolution, they do not retain the other major benefit of growth coupling, i.e., the maintenance of strain performance in a new environment, notably, the large-scale bioreactor.

Aside from the difficulty in generalizing the growth coupling strategy, the major drawback is that a substantial amount of carbon is diverted to biomass. The alternative is a process in which the growth and production stages are separated, typically by limiting a necessary nutrient.^128,129^ However, overall metabolic rates may be drastically lowered^130^ as there is little selection pressure for microbes to maintain active metabolism in the absence of growth. Understanding this regulation and engineering the decoupling of growth and metabolism is a major challenge in the field.

## Scaling up

### Physiology and metabolism

A grand challenge in metabolic engineering is scaling production from typical lab conditions (1–100 ml volumes and relatively low substrate and biomass concentrations) to commercial reactors (100–10^6^ l volumes, high cell density and substrate loading) without losing performance.^18^ Average process development typically takes 5–10 years and is significantly more expensive than scaling up an equivalent chemical process.^131^ Typically, initial attempts lead to reduced yields, undesired side products, and ultimately a diminished batch-to-batch consistency and product quality, with important economic consequences.^132^

Many possible factors can account for this drop in performance. Although initial strain testing is done in batch cultures, the fed-batch process used at larger scale leads to different physiology and metabolic states, which may no longer provide the necessary flux distribution in central metabolism. A longer growth period may select for strains that avoid the fitness cost of diverting large amounts of carbon and energy to the product. The strategies outlined above, to either couple production to growth, or separate the growth and production stages, are critical to success. For strain improvement beyond the initial pathway characterization, it is critical to consider process parameters, and design small-scale testing procedures that mimic the large-scale process as much as possible.^131^ In this regard, a widely used approach is to keep certain dimensionless co-efficients (such as dimensionless mixing time, or power input number^132^) constant. Probably the most applied scale-up variable is the volumetric oxygen transfer coefficient (k_L_a), characterizing the oxygen availability.

### The volume effect

One aspect of the scale-up process that is challenging to mimic at laboratory scale is the direct effect of reactor volume and associated inhomogeneity in the bioreactor. In typical bioreactor setups, inefficient mixing produces gradients of glucose and oxygen, which can create deviations as large as 400-fold and reduce biomass yield by as much as 20%.^133^ When similar inhomogeneities were reproduced in a smaller bioreactor for a *S. cerevisiae* fermentation, the aerobic/anaerobic shifts increased by-product formation, reduced biomass growth, and increased oxygen demand.^134,135^ Similarly, large-scale *E. coli* studies revealed increased acetate formation and reduced biomass growth as compared with lab-scale fermentations.^131,133^ More interestingly, Ying Lin *et al.* ^136^ showed reduced heterologous protein formation for *E. coli* in an oscillating glucose supply, which also resulted in increased carbon dioxide production. Furthermore, transcriptional studies in *E. coli* under shifting aerobic/anaerobic conditions showed a 1.5- to 6-fold increase in transcript levels for mixed acid fermentation genes and several global regulators.^137^

**Figure 3:**
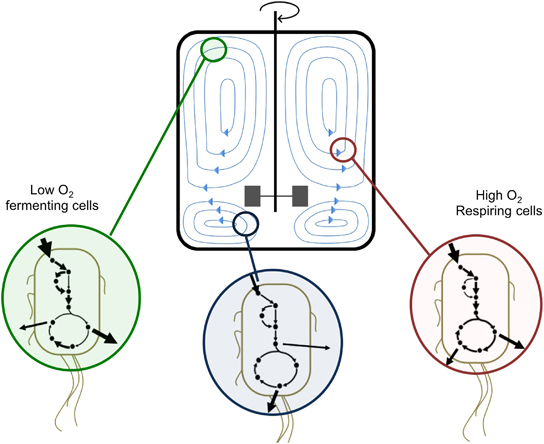
Integrated Fluid Dynamics (IFD) merges traditional fluid dynamics analysis describing fluid flow (blue lines in the bioreactor on top of the figure) and oxygen and substrate profiles, with models of bacterial metabolism. A significant challenge consists in coupling IFD with metabolic models that are as accurate and sophisticated as those describing the physicochemical characteristics of the fermentation.

Understanding and predicting the volume-related performance changes in large-scale fermentation usually consists of combining the information from down-scaling experiments with fluid dynamics simulations of the physical and chemical conditions in the bioreactor.^138^ This combination has been carried out by coupling kinetic models of metabolism with fluid dynamics simulations^139,140^ (Figure 3). However, while fluid dynamics simulations are rather sophisticated in predicting fluid flow, glucose, and dissolved oxygen profiles,^140,141^ the metabolic models coupled with them are not as sophisticated (e.g., six differential equations for the kinetic model in Lapin *et al.* ^141^). This leads to a widespread opinion^131,142^ that more accurate modeling of microbial metabolism based on data gathered in scale-down experiments is required for effectively predicting performance at, e.g., 100 l from data obtained at a 35 ml shaking flask. This integration remains an open problem in the field that, if solved, would facilitate the commercialization of the products of metabolically engineered strains.

### Toxicity of final products

Another common failure mode of scale-up is the toxicity associated with the higher titer of the final product. Main categories of commodity compounds being targeted in metabolic engineering include fuels, fuel additives, precursors of polymers, plastics, materials, lubricants and adhesives, surfactants and solvents. C2–C12 compounds in these categories often have characteristics of hydrophobic solvents with octanol:water partition co-efficients^143^ that range from 1–4. Most microorganisms, even solventogentic microbes such as clostridium,^144^ are extremely sensitive to solvents in this range.^145–147^ A large number of systems biology studies have been used to explore the underlying causes of toxicity, spanning from transcript and proteome analysis in several different microbes (e.g., *E. coli*, clostridia, pseudomonads, and cyanobacteria)^47,67,148^ to MAGE, recombineering, and genome-wide fitness screens. Comprehensively reviewed elsewhere,^47,149–153^ these studies aim to identify candidates that can then be used to improve the tolerance characteristics.^154,155^ Screening heterologous gene libraries or fosmid libraries from other microbes with desirable functions is also an effective method to obtain genes that provide such phenotypes and has been used to discover novel efflux pumps from organisms that provided improved tolerance and increased production for monoterpenes in *E. coli,* ^156^ and to mitigate toxicity from by-products of biomass pretreatments.^72,157^

## Systems biology for synthetic biology: gaps and challenges

The combination of the unprecedented capabilities afforded by synthetic biology with the comprehensive description of biological entities provided by systems biology presents a unique range of opportunities in designing and understanding biology. The recent technical advances in next-generation sequencing, high sensitivity proteomic and metabolomic methods, and developments in fluxomic techniques make systems biology methods more powerful and accessible to the synthetic biology community. The opportunity to use these methods to inform both synthetic biology tool development and metabolic engineering efforts makes significant scientific discoveries possible. The successes described above form a foundation for systems-level development of a wide variety of synthetic biology approaches. Comprehensive genome engineering for tolerance or target molecule production directly benefits from sequencing methods for strain characterization leading to rapid progress toward the goal of developing robust microbial cell factories. However, despite many successes, exciting new techniques or systems that work beautifully in the laboratory often do not directly scale to industrial fermentation conditions, or are not robust to minor changes in system parameters. Efforts to standardize and report experimental conditions have greatly improved the power of transcriptomic data sets, yet the same efforts have not permeated the proteomic, metabolomic, or fluxomic worlds to a comparable degree. Furthermore, the low-sample throughput of these -omics methods severely limits the usefulness of the data beyond the hypotheses of direct interest. Consequently, both systems and synthetic biology would benefit from research efforts that emphasize reproducibility, method and data sharing, and increased throughput of proteomic, metabolomic, and fluxomic analyses.^158^

Limitations in reproducibility hamper predictability. A process that is not quantitatively reproducible or exhibits a large unknown variability can hardly be used to parameterize predictive models, and cannot be expected to perform as needed under the required conditions. This inability to predict the behavior of biological systems under conditions not experimentally studied lies at the base of our inability to predict the behavior of large-scale fermentations.^159^ Whereas we can model and predict the physical side of chemical engineering (e.g., fluid flows, dissolved oxygen profiles), our capability to predict biological systems lags behind, hindering our capability to assure proper functioning under non-studied conditions.

Greater reproducibility and predictability is enabled by efficient data and metadata collection and sharing. One cannot fully compare local experiments with similar ones performed in different laboratories without a detailed account of the conditions and reagents. Concurrently, computational biologists, modelers and data scientists worldwide are limited by experimental data with which to validate a growing number of computational approaches designed to predict biological behavior.^107,160,161^ However, few laboratories perform experimental and computational work simultaneously because of the difficulties of doing so. Hence, the collaboration between experimentalist and computational specialists could become much more fruitful and frequent through a more robust exchange of data. Several initiatives tackle this problem,^162–164^ but there is more work to be done to enable effective and fast data exchange. These data exchange are particularly important for the further development of synthetic biology and biological computer aided design:^158^ as has been shown by Davidsohn *et al.,* ^165^ the rigorous characterization of pathway parts enables the accurate prediction of the behavior of full pathways.

### Conclusion

The uses of synthetic biology showcase the extraordinary opportunities in what has been called the century of biology,^166^ from understanding fundamental scientific questions to unique practical applications. Once biological systems are pushed to their limits (in terms of, e.g., product yield improvement), systems biology becomes fundamental to understand and predict them. However, bioengineering takes significantly more time and effort and is much less precise than other types of engineering in more established fields (civil, mechanical, electrical, etc). Changing the status quo will require significant investments in the basic tools that will improve productivity and precision in biological design, just as, e.g., integrated circuits have for the computer sciences. In the information technologies field, implementation has been optimized to the point that a limited amount of money and a small team is sufficient to create enough value to launch a company. The situation is the opposite in biological engineering, where implementation of ideas takes much more effort than creating them. In this review, we have presented what we believe are some challenges that need to be overcome in order to reach that level of maturity.
